# Case Report: Bladder adenocarcinoma: primary or urachal?

**DOI:** 10.12688/f1000research.20106.1

**Published:** 2019-10-04

**Authors:** J. Eduardo Tejeda-Mariaca, Marco Ordoñez-Alcantara, Aldo Bello-Sedano, Victor Perez-Cornejo, J. Antonio Grandez-Urbina

**Affiliations:** 1 Urology Deparment, Hospital Nacional Alberto Sabogal Sologuren, Callao, 07001, Peru; 2 Uroscience Research Group, Lima, 15046, Peru; 3 Universidad Nacional Mayor de San Marcos, Lima, Peru; 4 Biomedical Research Institute, Universidad Ricardo Palma, Santiago de Surco, Lima, 15039, Peru; 5 Universidad Continental, Miraflores, Lima, 15046, Peru

**Keywords:** Urinary Bladder Neoplasms, Adenocarcinoma, Surgical Pathology

## Abstract

**Background:** Bladder adenocarcinoma (AC) is a scarce histological variant and there are few studies on its proper management. No previous case reports present the management of a urachal tumor and the incidental finding of bladder adenocarcinoma.

**Clinical case:** We present the case of a young woman with nonspecific symptoms, who presented with a prior history of dysuria, bladder tenesmus, suprapubic pain and urinary urgency for one year, which had been treated as recurrent urinary tract infection. A partial cystectomy plus extended lymphadenectomy was scheduled. We found a bladder tumor with characteristics of a urachal tumor and the pathological report indicated a primary bladder AC. The patient had a complete recovery at one year of follow-up.

**Conclusions:** A patient can present with a tumor with urachal characteristics; however, the pathology report can show primary AC. The decision to perform partial cystectomy was an appropriate option for the location of this tumor, with optimal surgical results. Still, a long-term follow-up is necessary. More specific management guidelines are required for the treatment of AC.

## Background

Within bladder tumors, adenocarcinoma (AC) is a histological variant that represents only 0.5–2% of cases. Its prognosis is the poorest, given that it is diagnosed in more advanced stages due to its rarity. There is little literature about its management and there are no standard treatment guidelines.

Its association with a history of bladder exstrophy, schistosomiasis and chronic bladder irritation has been described. Due to intramural growth, the symptoms occur later in disease progression and AC is diagnosed in more advanced stages, so the prognosis is worse. Only 5% of cases are diagnosed during the initial stages. Hematuria is the most frequent symptom (60–100% of cases), and irritative symptoms and mucosuria are also common (25–80% of cases) ^1^.

Bladder AC can be classified as primary or secondary, the latter occurring by direct extension or by metastasis from a distant site like the colon, prostate, endometrium, cervix or breast. Strictly, the urachus is not an intrinsic component of the bladder. However, urachal AC is usually described with bladder tumors because they share pathological and clinical features. Therefore, bladder AC can be classified as urachal AC (10–30%) and non-urachal AC (70–90%).

Primary bladder AC shows a pure glandular phenotype. It usually arises from the trigone and the posterior wall but can be found anywhere in the bladder. It usually presents as a solitary lesion ^2^. Histologically, it shows several growth patterns: enteric, morphologically identical to its colonic counterpart; or mucinous, with abundant extravasated mucin, including signet ring cells.

Urachal AC develops from the remnant of urachus. It presents as a solitary polypoid mass in the dome of the bladder, although it can be seen anywhere along the anterior midline, and it can affect the Retzius space and the anterior abdominal wall. Microscopically, it is very similar to primary AC, the mucinous variant being the most frequent.

Here, we report an incidental case of a patient with bladder AC treated as urachal AC who presented good oncological results at one year of follow-up. What is unique about this case is that urachal AC tumor management was proposed because of the clinical findings; however, the urachus was ultimately found to be tumor-free.

## Clinical case

### Patient information

**Figure 1.**
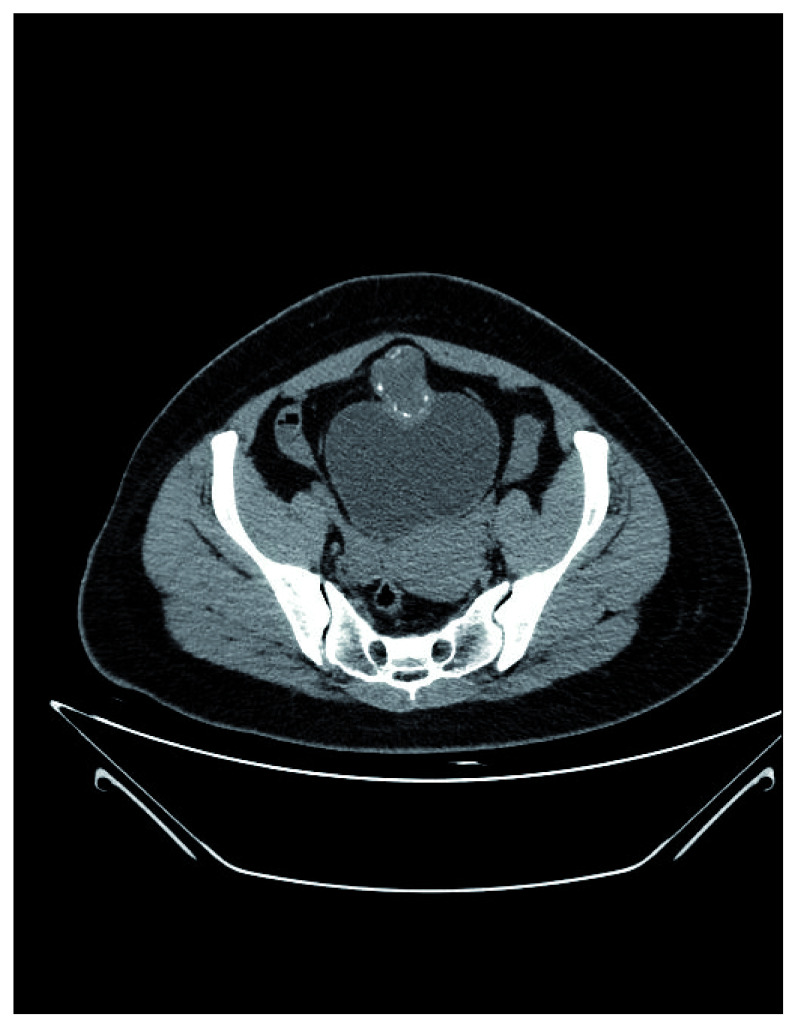
Lower abdominal CT scan showing superior and anterior bladder mass and cystic lesions with peripheral calcifications.

The patient was a 35-year-old mestizo woman, who works as a junior manager, with no clinical history of hematuria, bladder tumors or prior surgical interventions and no family history of bladder tumors. The patient presented to the urology practice with a prior history of dysuria, bladder tenesmus, suprapubic pain and urinary urgency for one year, which had been treated as recurrent urinary tract infection with broad spectrum antibiotics. The patient presented negative cultures; however, the symptoms did not disappear. A timeline of the major timepoints in the patient’s history, diagnosis and treatment is provided ( Figure 1).

### Clinical findings

A physical abdominal and genitourinary exploration was carried out. There were no positive findings upon physical examination and no painful trigger points were found. There were no signs of vulvar irritation or palpable abdominal mass.

### Diagnostic assessment

**Figure 2.**
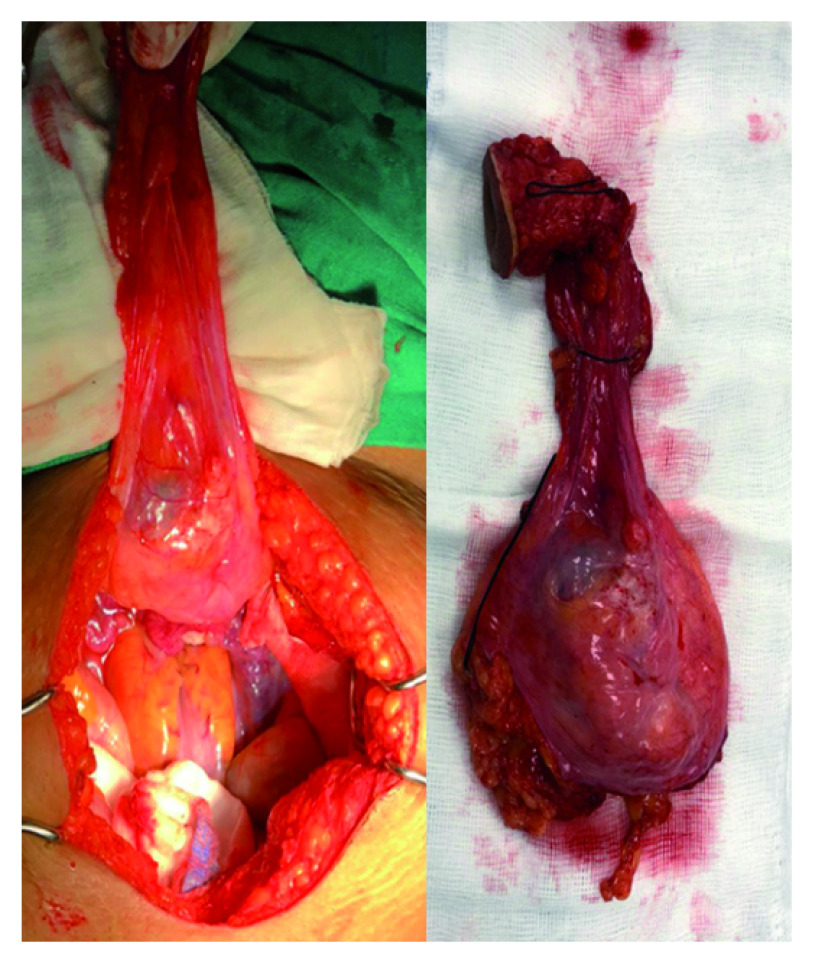
Partial cystectomy with urachal resection plus omphalectomy. A bladder dome mass of approximately 5cm was resected.

A bladder screening ultrasound was performed in order to identify any abnormal structure or urinary retention. During the exam the bladder was full, and a bladder dome mass was noted. A unique, polypoid mass with mucoid characteristics of 4.0cm was found using urethrocytoscopy. A lower abdomen contrasted CT scan was performed and a collection/mass was located on the anterior and superior edge of the bladder of 60 by 40mm, which was cystic and solid (density of 30UH) and had peripheral calcifications ( Figure 2). Following this, a transurethral resection was performed. In the transurethral resection pathological report, moderately differentiated muscle invasive mucinous AC was reported. Taking into account these findings, an endoscopy, colonoscopy and mammography were performed, but there was no evidence of tumor in the exams. A solid or cystic mass in the midline with calcifications is considered a major finding indicative of urachal AC and so the diagnosis of urachal AC was proposed.

### Therapeutic interventions

**Figure 3.**
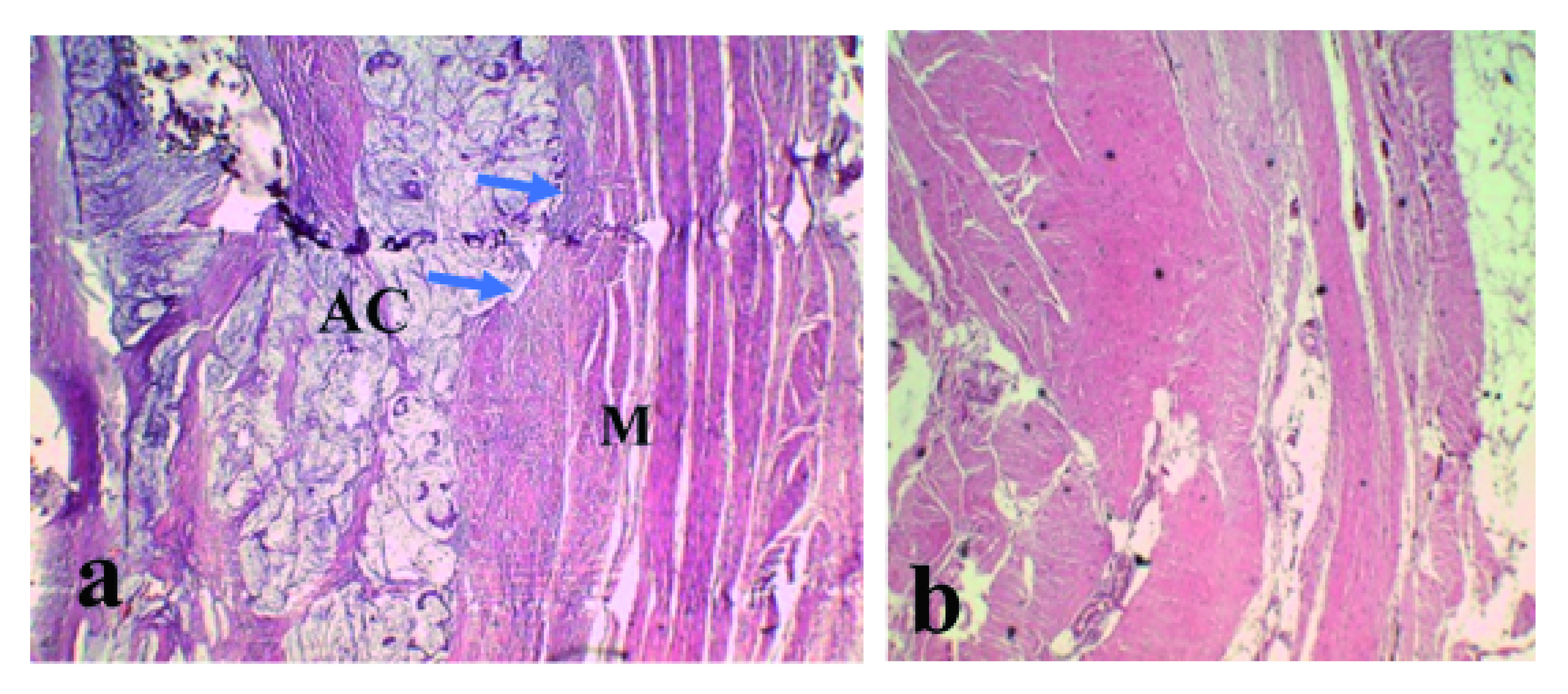
**a**) Primary adenocarcinoma (AC) and infiltrated muscle layer (M) (blue arrows). **b**) Tumor-free urachus.

Mobile solitary tumors that are away from the base may potentially benefit from partial cystectomy, so a partial cystectomy plus extended lymphadenectomy was scheduled ( Figure 3). There were no pre-intervention considerations. The patient was placed in dorsal decubitus position and spinal anesthesia plus epidural catheter, with bupivacaine hydrochloride at 0.5%, without adrenaline and without preservatives, was administered without any complications.

The surgical intervention was performed by an experienced surgeon without complications. A bladder dome mass of approximately 5cm was resected. In the partial cystectomy pathology report, an invasion of the proximal third of muscle layer was described. Clear surgical margins were reported, and no positive lymphatic nodules were found. There was no evidence of infiltration in the area corresponding to the remnant of urachus. Immunohistochemical analysis showed the tumor tested positive for Cytokeratin 20 (CK20) and Cytokeratin 7 (CK7) that are distributed in epithelia and their neoplasms. However, the test for carcinoembryonic antigen (CEA), which is a marker of colon carcinoma cells, was negative.

**Figure 4.**
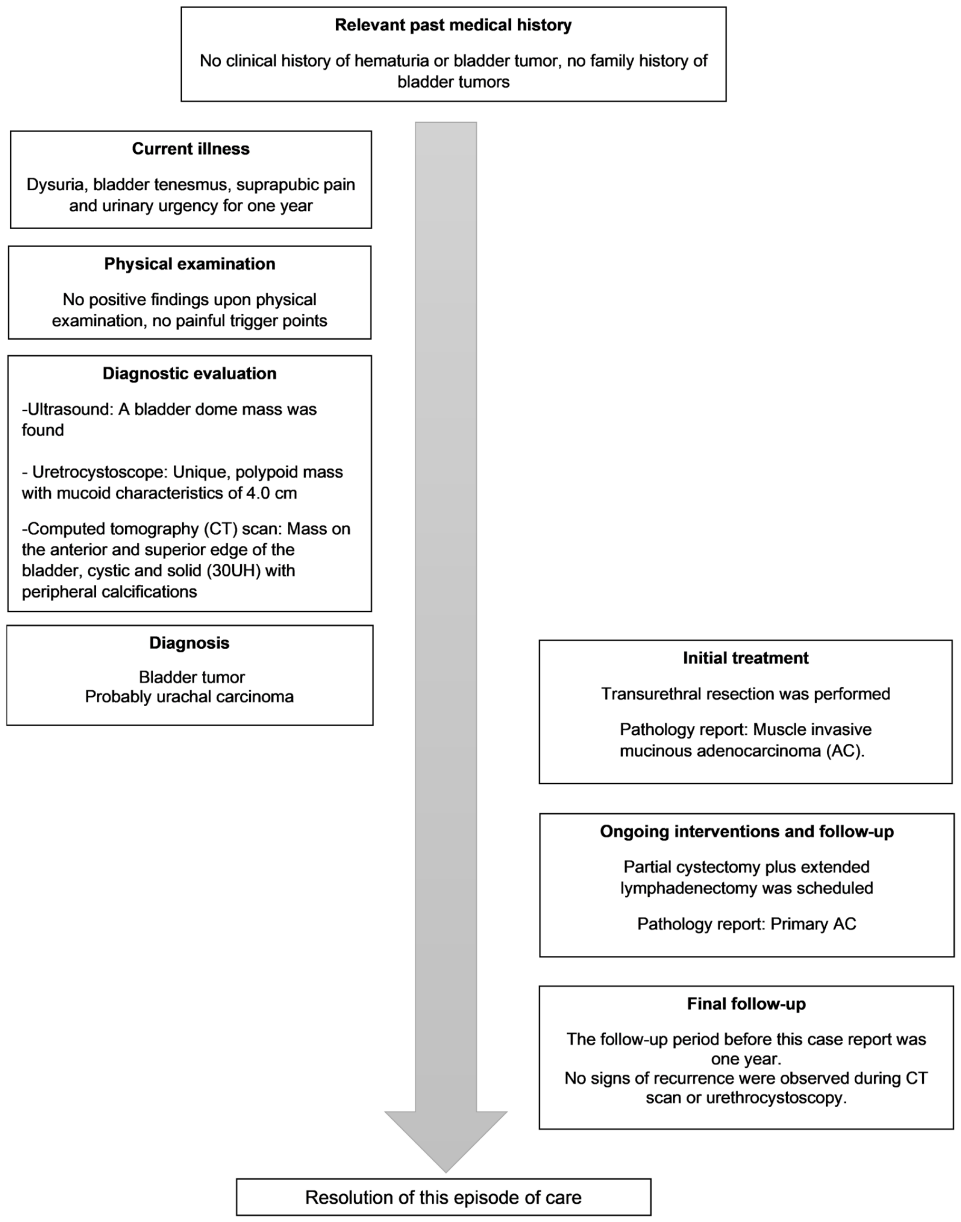
Timeline of major timepoints in the patient’s history, diagnosis and treatment.

The urachus was tumor-free ( Figure 4). However, the bladder layer presented a tumor in its dome without any evidence of secondary AC. Therefore, the final diagnosis was primary bladder AC.

### Follow-up and outcomes

The patient was discharged 10 days after the surgical intervention. Cephalexin 500mg three times a day was prescribed for five days after discharge. The Foley catheter was removed 14 days after surgery. No complications and no urinary fistula were reported. No chemotherapy was administered. No signs of recurrence were observed during a CT scan and urethrocystoscopy performed after a follow-up period of one year.

## Discussion

Although the bladder is not a common site of metastasis, secondary AC is more frequent than primary AC. Cancer cells can spread by direct extension or by the hematogenous/lymphatic route. During diagnosis, ultrasound is useful as an initial imaging test; however, CT scanning and MRI provide solid information to determine the extent of the disease, rule out metastases and evaluate if it is potentially resectable. In our case report there was evidence in the CT scan that a collection/mass was located on the anterior and superior edge of the bladder; however, it may have been interesting perform an MRI in order to precisely identify any urachal involvement.

A mass in the midline, solid or cystic, with calcifications is considered a major finding indicative of urachal AC. Cystoscopy and transurethral resection of the tumor confirms the diagnosis. Peritoneal carcinomatosis, as well as peritoneal pseudomyxoma, can be a finding in patients with metastatic disease. The analysis of CEA, CA125 and CA19-9 antigen levels should be carried out, which may be elevated in 40%–60% of these patients. The diagnosis of primary bladder AC should be made only after the exclusion of a secondary AC. Therefore, it is necessary to perform colonoscopy, endoscopy, mammography and colposcopy. The histopathological findings are difficult to use to differentiate between the types of AC and immunohistochemistry has limited utility for the differential diagnosis. The diversity of AC means that cytological preparations are a challenge because immunohistochemistry has limited utility.

The low frequency of AC and the absence of large studies explain the absence of clearly established therapeutic guidelines. In primary AC, radical cystectomy and dissection of pelvic lymph nodes are the first option. However, mobile solitary tumors that are away from the base may potentially benefit from partial cystectomy ^1^. In urachal AC, partial cystectomy is the standard procedure, with block resection of the bladder dome, urachal ligament, and umbilicus ^3, 4^. Lymphadenectomy (LD) is necessary when the incidence of lymph node metastasis in AC is high at the time of diagnosis. LD improves survival, time before recurrence and staging. Therefore, performing extended LD would be the most appropriate option in these patients ^5^.

The role of chemotherapy is not yet clear. However, some cohort studies have shown benefit in high-risk patients (advanced stage, positive margins, positive nodes). This is based on cisplatin and 5-fluorouracil ^4^. The use of radiotherapy is also not clear in bladder AC. Some studies showed better oncological results with positive nodes and recurrence. Despite this, its advantage in terms of oncological results has not been established with adequate studies. It can be recommended for local control only ^6^.

## Conclusions

1. A patient can present with a tumor with urachal characteristics, however, the pathology report can show primary AC.
2. The decision to perform partial cystectomy was an appropriate option for the location of this tumor, with optimal surgical results. Still, a long-term follow-up is necessary.
3. More specific management guidelines are required for AC.

## Data availability

All data underlying the results are available as part of the article and no additional source data are required.

## Consent

Written informed consent for publication of their clinical details and clinical images was obtained from the patient.
