# Supplementary material for: Case Report: Bladder adenocarcinoma: primary or urachal?
Source: F1000Res. 2019 Oct 4;8:1717. [Version 1] doi: 10.12688/f1000research.20106.1 (PMC7416577; doi:10.12688/f1000research.20106.1)
Supplement: Peer review history

# Peer review: Reviewer response for version 1

**DOI:** 10.5256/f1000research.22074.r67951

**Published:** 2020-08-12

**Authors:** Alejandro Carvajal Obando

**Affiliations:** 1 Universidad CES, Medellín, Colombia

It is an interesting work and an unusual find in that geography. It is valuable in keeping urologists aware of the possibility of such a differential diagnosis.

 However, I make several clarifications:

1. The abstract is not clear, it is not understood why the surgical treatment is noted (partial cystectomy) and the full text must be read to understand it
2. During the discussion, they focused little on the pathophysiology and epidemiology of the type of tumor, I think it should be expanded further because they discussed the diagnosis and treatment options.
3. ​​​​The conclusions of the work should not justify the findings of the case, but rather contextualize the analysis of the case 

Are enough details provided of any physical examination and diagnostic tests, treatment given and outcomes?

Yes

Is the case presented with sufficient detail to be useful for other practitioners?

Partly

Is sufficient discussion included of the importance of the findings and their relevance to future understanding of disease processes, diagnosis or treatment?

Partly

Is the background of the case’s history and progression described in sufficient detail?

Yes

Reviewer Expertise:

General Urology, Andrology, MAle Infertility

I confirm that I have read this submission and believe that I have an appropriate level of expertise to confirm that it is of an acceptable scientific standard, however I have significant reservations, as outlined above.

# Peer review: Reviewer response for version 1

**DOI:** 10.5256/f1000research.22074.r67953

**Published:** 2020-08-07

**Authors:** Oriana Rivera

**Affiliations:** 1 Vicerrectorado de Investigación, Universidad Norbert Wiener, Lima, Peru

- The article is well structured and fulfills one of the educational functions.
- The introduction expresses the reason why it is intended to publish, there is a review of the topic and explains the relevance of the topic.
- The case description has important antecedents, the interrogation, the physical examination, the diagnostic support studies, and the results.
- In the discussion, the authors emphasize why the case is remarkable and explains or clarifies the important aspects and compares themselves with other students.

In conclusion, the document is suitable for indexing.

Are enough details provided of any physical examination and diagnostic tests, treatment given and outcomes?

Yes

Is the case presented with sufficient detail to be useful for other practitioners?

Yes

Is sufficient discussion included of the importance of the findings and their relevance to future understanding of disease processes, diagnosis or treatment?

Yes

Is the background of the case’s history and progression described in sufficient detail?

Yes

Reviewer Expertise:

EPIDEMIOLOGY AND PUBLIC HEALTH

I confirm that I have read this submission and believe that I have an appropriate level of expertise to confirm that it is of an acceptable scientific standard.

# Peer review: Reviewer response for version 1

**DOI:** 10.5256/f1000research.22074.r65012

**Published:** 2020-07-06

**Authors:** Marlon Perera

**Affiliations:** 1 Department of Urology, Princess Alexandra Hospital, Woolloongabba, Qld, Australia

The authors provide a case report on the management of muscle-invasive adenocarcinoma of the bladder with partial cystectomy. As discussed by the authors, this is not standard or recommended treatment for this disease. This treatment strategy has been well addressed in the literature.

**Background:**

- Schistosomiasis and chronic bladder irritation are associated with squamous cell carcinoma of the bladder (not adenocarcinoma).

**Case report: **

- It is unclear the duration of followup. It would be useful to know longer-term oncologic outcomes. Specifically, the authors are advocating for partial cystectomy in T2 adenocarcinoma of the bladder without a clear report of the oncologic outcome.

I'm not entirely sure of the novelty of this case and its' suitability for publication.

Are enough details provided of any physical examination and diagnostic tests, treatment given and outcomes?

Yes

Is the case presented with sufficient detail to be useful for other practitioners?

No

Is sufficient discussion included of the importance of the findings and their relevance to future understanding of disease processes, diagnosis or treatment?

No

Is the background of the case’s history and progression described in sufficient detail?

Yes

Reviewer Expertise:

Urology, urooncology

I confirm that I have read this submission and believe that I have an appropriate level of expertise to state that I do not consider it to be of an acceptable scientific standard, for reasons outlined above.

# Peer review: Reviewer response for version 1

**DOI:** 10.5256/f1000research.22074.r59590

**Published:** 2020-02-21

**Authors:** Orsolya Módos

**Affiliations:** 1 Department of Urology, Semmelweis University, Budapest, Hungary

This report presents a case of a young woman with adenocarcinoma presented in the bladder. With endoscopic procedures, secondary involvement of the bladder was excluded. Based on imaging findings the presence of urachal carcinoma was supposed, therefore partial cystectomy with extended lymphaedenctomy and the removal of the umbilical remnant and the umbilicus was performed. The pathological examination of the tumor found primer bladder adenocarcinoma.

 The case report is well written, the train of thought is traceable, but there are some diagnostic tools which could suggest the presence of urachal cancer before open surgical treatment. Therefore I have some minor point to discuss: 

1. Serum tumor markers as CEA, CA-19-9, CA125 and CA-724 can be elevated in urachal cancer. ^1^^,^^2^ What was the level of these serum tumor markers before and after partial cystectomy?
2.  However, there is no reliable immunohistochemical marker which can distinguish between urachal and primary bladder adenocarcinoma, some of them can suspect the origin of the examined adenocarcinoma. Were immunohistochemical examinations performed of the TURB tumor sample (e.g. AMACR, CK34bE12, GATA3)? This should be mentioned in the manuscript.
3. Histological examination of partial cystectomy resulted primary bladder adenocarcinoma. What was the TNM-stage of this?
4. In the case of primary bladder adenocarcinoma, radical cystectomy is suggested. In this case, urachal cancer was supposed before surgical treatment, therefore partial cystectomy was performed, however the final diagnosis was primary adenocarcinoma. In the future, is radical cystectomy proposed and if so, when?  

Are enough details provided of any physical examination and diagnostic tests, treatment given and outcomes?

Partly

Is the case presented with sufficient detail to be useful for other practitioners?

Yes

Is sufficient discussion included of the importance of the findings and their relevance to future understanding of disease processes, diagnosis or treatment?

Yes

Is the background of the case’s history and progression described in sufficient detail?

Yes

Reviewer Expertise:

urachal cancer, urological cancer

I confirm that I have read this submission and believe that I have an appropriate level of expertise to confirm that it is of an acceptable scientific standard.

## References

[1] : Genetics and biological markers in urachal cancer. *Transl Androl Urol*.2016;5(5) : 10.21037/tau.2016.04.01 655-661 10.21037/tau.2016.04.01 27785422PMC5071183

[2] : Biomarkers in Urachal Cancer and Adenocarcinomas in the Bladder: A Comprehensive Review Supplemented by Own Data. *Dis Markers*.2018;2018: 10.1155/2018/7308168 7308168 10.1155/2018/7308168 29721106PMC5867586
